# Ultrasound Imaging as a Visual Biofeedback Tool in Rehabilitation: An Updated Systematic Review

**DOI:** 10.3390/ijerph18147554

**Published:** 2021-07-15

**Authors:** Juan Antonio Valera-Calero, César Fernández-de-las-Peñas, Umut Varol, Ricardo Ortega-Santiago, Gracia María Gallego-Sendarrubias, José Luis Arias-Buría

**Affiliations:** 1 Department of Physiotherapy, Faculty of Health, Universidad Camilo José Cela, 28692 Villanueva de la Cañada, Spain; gmgallego@ucjc.edu; 2 Department of Physical Therapy, Occupational Therapy, Rehabilitation and Physical Medicine, Universidad Rey Juan Carlos, 28922 Alcorcón, Spain; cesar.fernandez@urjc.es (C.F.-d.-l.-P.); ricardo.ortega@urjc.es (R.O.-S.); joseluis.arias@urjc.es (J.L.A.-B.); 3 Cátedra Institucional en Docencia, Clínica e Investigación en Fisioterapia: Terapia Manual, Punción Seca y Ejercicio Terapéutico, Universidad Rey Juan Carlos, 28922 Alcorcón, Spain; 4 IE School of Human Sciences and Technology, IE University, 28006 Madrid, Spain; umut.varol@alumni.ie.edu

**Keywords:** ultrasound imaging, rehabilitation, feedback, motor control, systematic review

## Abstract

Rehabilitative ultrasound imaging (RUSI) is used by physical therapists as a feedback tool for measuring changes in muscle morphology during therapeutic interventions such as motor control exercises (MCE). However, a structured overview of its efficacy is lacking. We aimed to systematically review the efficacy of RUSI for improving MCE programs compared with no feedback and other feedback methods. MEDLINE, PubMed, SCOPUS and Web of Science databases were searched for studies evaluating efficacy data of RUSI to improve muscular morphology, quality, and/or function of skeletal muscles and MCE success. Eleven studies analyzing RUSI feedback during MCE were included. Most studies showed acceptable methodological quality. Seven studies assessed abdominal wall muscles, one assessed pelvic floor muscles, one serratus anterior muscle, and two lumbar multifidi. Eight studies involved healthy subjects and three studies clinical populations. Eight studies assessed muscle thickness and pressure differences during MCE, two assessed the number of trials needed to successfully perform MCE, three assessed the retain success, seven assessed the muscle activity with electromyography and one assessed clinical severity outcomes. Visual RUSI feedback seems to be more effective than tactile and/or verbal biofeedback for improving MCE performance and retention success, but no differences with pressure unit biofeedback were found.

## 1. Introduction

Motor control exercise (MCE) consists of an exercise-based intervention focused on the activation of deep muscles to improve the control and coordination of these muscles [1]. MCE is widely used since evidence suggests improvements in pain, function, self-perceived recovery and quality of life up to 12 weeks [1]. Several mechanisms, including the lack of stability of the spine, impaired motor control and/or muscle activity patterns, or disturbed proprioception and restricted range of motion, have been proposed for explaining non-specific spine pain [2]. Motor control exercises aim to restore muscular coordination, control and capacity by training isolated contractions of deep trunk muscles while maintaining a normal breathing and progressing to pre-activate and maintain the contraction during dynamic and functional tasks [3]. Given the difficulty that some patients can perceive during MCE, these exercises are usually performed in supervised sessions providing biofeedback on the activation of trunk muscles for facilitating the awareness and control of these deep muscles’ isolated contractions [4].

According to the definition provided by Blumenstein et al. [5], biofeedback refers to external psychological, physical, or augmented proprioceptive feedback that is used to increase an individual’s cognition of what is occurring physiologically in the body. Although several modalities are described in the literature (e.g., electroencephalography, skin resistance, electrocardiography, sphygmomanometry, strain-gauge devices, thermal feedback), the most used biofeedback modalities include ultrasound imaging, pressure biofeedback units and electromyography.

Ultrasound imaging (US) is a fast, easy, safe, noninvasive and low-cost real-time method frequently used for assessing muscle morphology (e.g., thickness, cross-sectional area and volume) [6], quality (e.g., echo-intensity and fatty infiltration) [7] and function [8]. This method allows both patients and clinicians to see in real time muscle morphology changes, since this is sensitive to positive and negative changes and therefore is valid for measuring trunk muscle activation during isometric submaximal contractions [9].

Surface electromyography, which consists of placing surface electrodes to detect changes in skeletal muscle activity for providing to the patient a visual or auditory signal for either increasing or reducing muscle activity, is also used as a biofeedback method in rehabilitation [10,11]. However, surface EMG cannot be used for assessing deep muscles and needle electrodes are needed [12].

Finally, pressure biofeedback units are also commonly used since they are economic and easy to apply in a clinical setting. This instrument consists of an inflatable cushion which is connected to a pressure gage, which displays feedback on muscle activity [13].

Since the last systematic review assessing the efficacy of Rehabilitative Ultrasound Imaging (RUSI) for enhancing the performance and contraction endurance of skeletal muscles during MCE was published more than 10 years ago and new evidence is available [14], an updated systematic review is needed. Thus, although a previous review by Giggins et al. [15] reviewed the biofeedback therapies used in rehabilitation, RUSI was not compared with others biofeedback methods nor without feedback. Therefore, the current systematic review evaluates the efficacy of RUSI to improve muscle function during CME compared with no feedback and other feedback methods in both healthy subjects and patients with musculoskeletal pain conditions.

## 2. Materials and Methods

### 2.1. Study Design

This systematic review adheres to the Preferred Reporting Items for Systematic Reviews and Meta-Analyses (PRISMA) statement [16]. The international OPS Registry registration link is https://doi.org/10.17605/OSF.IO/CNGW4 (accessed on 15 February 2021).

### 2.2. Data Sources

Since a minimum of three databases are needed for adequate systematic reviews [17], we conducted a search in the following electronic literature: MEDLINE, PubMed, SCOPUS and Web of Science databases from their inception to 18 February 2021. Search strategies were conducted with the assistance of an experienced health science librarian and following the guidelines described by Greenhalgh [18]. Search strategies were based on a combination of MeSH terms and key words following the PICO (Population, Intervention, Comparison, Outcome) question:

Population: Adults (older than 18 years old) with or without musculoskeletal pain disease.

Intervention: Use of real-time ultrasound imaging as visual biofeedback during MCE to facilitate the MCE performance or retention success.

Comparator: No biofeedback or other biofeedback method.

Outcomes: Improvements in muscular function as assessed with imaging methods (US, magnetic resonance imaging or computed tomography) or EMG.

An example of the search strategy (PubMed database) was as follows:
Filters: [Title/Abstract]#1 Ultrasonography [Mesh]: #2 Ultrasound; #3 Echography; #4 Sonography#5 #1 OR #2 OR #3 OR #4#6 Exercise Therapy [Mesh]: #7 Motor control; #8 Stabilization exercise; #9 Rehabilitation Exercise#10 #6 OR #7 OR #8 OR #9#11 Feedback, Sensory [Mesh]: #12 Biofeedback; #13 Visual Feedback; # 14 Audio Feedback; #15 Proprioceptive Feedback; #16 Sensorimotor Feedback#17 #11 OR #12 OR #13 OR #14 OR #15 OR #16# 18 Muscle, Skeletal [Mesh]#19 #5 AND #10 AND #17 AND #18

### 2.3. Study Eligibility Criteria

Experimental studies were eligible for inclusion if they (1) evaluated the efficacy of RUSI as visual feedback compared with any other feedback method; (2) used of RUSI for improving muscle function (either as performance or retaining success) of skeletal muscles; (3) included healthy subjects or symptomatic populations, and, (4) were published in English language. Animal studies, observational studies, descriptive studies, review studies, cadaveric studies, published proceedings, and abstracts were excluded.

### 2.4. Study Appraisal and Synthesis Methods

The Mendeley Desktop v.1.19.4 for Mac OS (Glyph & Cog, LLC 2008) program was used to insert the search hits from the databases. First, those duplicated studies were removed. Second, title and abstracts of the articles were screened for potential eligibility by two reviewers. Third, the full text was analyzed to identify potentially eligible studies. Both reviewers were required to achieve a consensus. If the consensus was not reached, a third reviewer participated in the process to reach the agreement for including or not including the study. A standardized data extraction form containing questions on sample population, methodology (intervention, comparator, tasks and muscle assessed), outcomes and results was used, according to the STARLITE guideline [19].

The methodological quality of the included studies was assessed using the PEDro scale [20]. This scale is used to assess the methodological quality of trials and consists of 11 items. The first item (not included in the total score) relates to external validity and the following 10 are used to calculate the final score evaluating the following features: random allocation, concealed allocation, similarity at baseline, subject blinding, therapist blinding, assessor blinding, lost follow-up, intention-to-treat analysis, between-group statistical comparison, and point and variability measures for at least one key outcome. Total PEDro scores between 0 and 3 are considered “poor”, 4 and 5 as “fair”, 6 and 8 as “good”, and 9 and 10 as “excellent” [20].

Finally, a risk of bias analysis for each study was conducted as recommended for systematic reviews [16]. The RoB 2 tool was used to identify the risk of bias in 5 domains: (1) bias due to randomization; (2) bias due to deviations from intended intervention; (3) bias due to missing data; (4) bias due to outcome measurement; and (5) bias due to selection of the reported result [21].

## 3. Results

### 3.1. Study Selection

**Figure 1:**
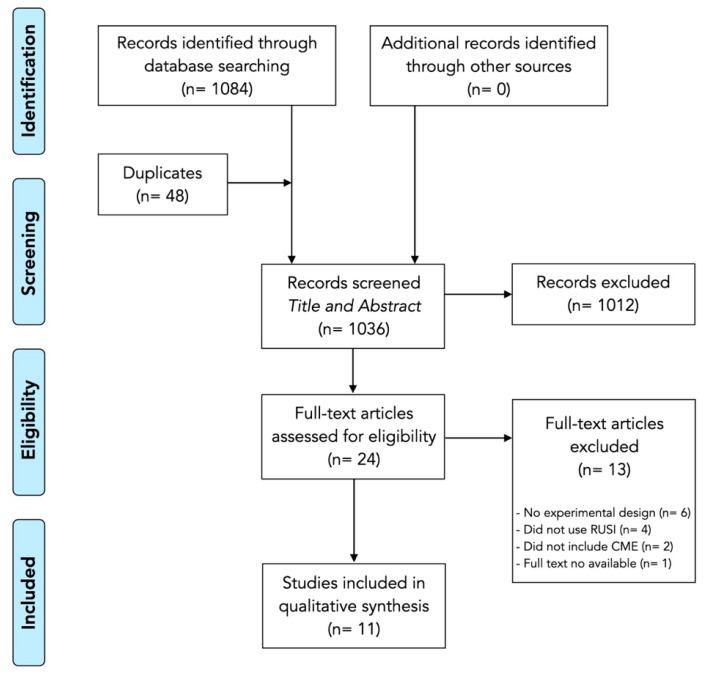
Preferred Reporting Items for Systematic reviews and Meta-Analyses (PRISMA) flowchart.

The results of the search and selection process (identification, screening, eligibility and analyzed) from the 1084 studies identified in the search to the 11 studies included in the review [22,23,24,25,26,27,28,29,30,31,32] are described in the flow diagram shown in Figure 1.

### 3.2. Methodological Quality and Risk of Bias

The methodological quality scores ranged from 4 to 9 (mean: 6.4, SD: 1.4) out of a maximum of 10 points (Table 1). The most consistent flaws were lack of participants (all studies) and therapist blinding (ten studies), concealed allocation (just five studies considered a concealed allocation) and providing point measures and measures of variability (eight studies).

**Figure 2:**
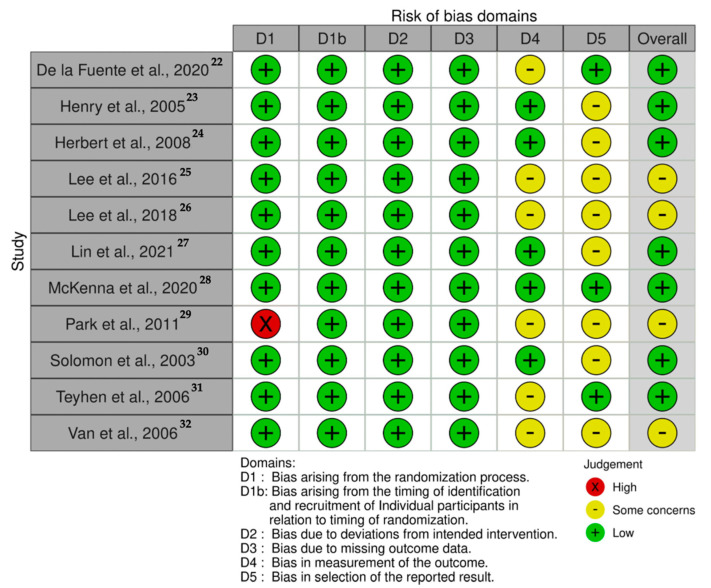
Risk of bias traffic-light plot.

The risk of bias analysis is described in Figure 2. Seven studies showed an overall low risk of bias [22,23,24,27,28,30,31]. However, four studies presented some concerns regarding the measurement of the outcomes and the reported results which should be considered on data interpretation [25,26,29,32].

### 3.3. Data Analysis

Table 2 summarizes the studies included in this systematic review investigating the efficacy of RUSI as biofeedback tool during MCE. The included studies compared RUSI visual feedback against verbal (*n* = 8) [22,23,25,26,27,29,31,32], tactile (*n* = 5) [23,25,28,30,31] and pressure unit (*n* = 2) [25,30] feedback. Further, one study evaluated different modalities of RUSI visual feedback (constant versus variable) [24].

Most studies assessed the deep abdominal wall musculature (including Transversus Abdominis -TrA- [22,23,25,26,27,29,31], Internal Oblique -IO- [23,25,26,29,31] and External Oblique -EO- [23,25,26,29,31]). Although procedures were not consistent (e.g., postures, measurement timing, resting between series, number of series, etc.), all studies assessing the abdominal wall muscles used the Abdominal Hollowing Exercise -AHE- [22,23,25,26,27,28,31]. In addition, pelvic floor muscles [30], serratus anterior [28] and lumbar multifidus -LM- [24,27,31] were also analyzed.

The included studies reported different outcomes since seven assessed changes in muscle thickness and/or pressure between MCE and rest [22,25,26,27,29,30,31,32], number of repetitions needed to correctly perform the MCE [22,23], ability to retain the correct MCE performance [23,24,31], muscle electromyographic activity [22,25,26,27,29,30,32], and clinical outcomes [30].

Regarding the populations included in the studies, most of them included healthy subjects [22,23,24,25,26,27,29,32] and just three studies included clinical populations, one study included patients with mild-to-moderate fecal incontinence [30], one study included patients with unilateral subacromial pain [28], and one study included patients with chronic low back pain [31]. In general, RUSI visual feedback was a more effective feedback tool than verbal feedback or single manual facilitation for most of the outcomes assessed (e.g., number of repetitions needed to perform correctly the MCE, muscle thickness, or electromyographic activity) considering that procedures were not consistent between studies. However, it seems equally effective as pressure biofeedback units.

## 4. Discussion

This systematic review found that RUSI applied as a visual biofeedback tool during MCE seems to be more effective for increasing muscle thickness, muscle activity and target exercise success when compared with verbal or tactile biofeedback. However, the results analyzed from the included studies suggest no additional benefit using RUSI when compared with pressure unit biofeedback. The studies included showed consistent flaws regarding their methodological quality, e.g., participant and therapist blinding, concealed allocation, point measures and measures of variability, which should be addressed in future studies.

To the best of the author’s knowledge, the last systematic review assessing the efficacy of RUSI for enhancing the performance and contraction endurance of skeletal muscles during MCE was published in 2007 and, therefore, findings from more recent evidence have not been previously updated [14]. Although our initial aim was to assess how RUSI could improve muscle function, muscular morphology, quality and/or function of skeletal muscles, most of the studies included healthy populations with neither decreased muscle quality nor decreased function. Therefore, although two studies included clinical pain populations, we cannot make definitive conclusions regarding the efficacy of RUSI for improving the mentioned outcomes.

Different comparative biofeedback methods were considered in studies included in this systematic review. Most of the studies included a common clinical biofeedback group (verbal biofeedback and/or tactile feedback) [22,23,25,26,27,29,31,32] and results seem to be consistent between trials. Comparative analyses showed larger changes in thickness [22,25,26,27,29,31,32], greater success for exercise performance (greater success ratio and lower number of trials to reach the first successful MCE performance) [23] and greater electromyographic activity [28] for the RUSI biofeedback groups, but no differences for MCE retention at short-term [23]. In the study conducted by Herbert et al. [24], constant (receiving real-time RUSI of successful or unsuccessful muscle activation on the monitor, but without verbal feedback) and variable (receiving delayed feedback after performing the exercise) RUSI feedback were compared. Although both methods sustained the MCE performance success at short-term, the constant feedback group showed superior motor learning at long-term.

Visual RUSI feedback was compared with pressure unit feedback in two studies [25,30]. The results seem to be consistent since Lee et al. [25] found that pressure unit feedback showed no differences for increasing muscle thickness compared with visual RUSI feedback and Solomon et al. [30] found similar improvements in MCE compliance, strength and clinical outcomes. Surprisingly, none of the studies included in this review compared RUSI feedback with other feedback methods (e.g., electromyography or sensitive stimulus). Although this study conducted by Vera et al. [4] was excluded since full-text is not available, their results showed no differences in muscular thickness change with or without sensitive electrical stimulation in addition to the visual RUSI biofeedback.

Although current evidence strongly supports the presence of motor control adaptations in patients with low back pain (LBP), including altered activation timings, lumbopelvic coordination, balance control and kinematics [32], and since MCE is a common form of exercise for LBP management, surprisingly we only identified two studies investigating the efficacy of RUSI in clinical populations (unilateral subacromial pain [28] and fecal incontinence [30]), but none included patients with LBP. Healthy population studies are not enough to conclude that visual RUSI biofeedback would obtain similar improvements in LBP populations for facilitating or improving muscular activity since these populations show brain plastic changes of the trunk musculature representation area [33], indicating less fine control [34]. It should be considered that, although MCE is an effective treatment for non-specific LBP, specially indicated for sub-clinical intermediate pain and middle-aged patients [35], low-to-moderate quality evidence showed no additional benefit over spinal manipulative therapy, other forms of exercise or medical treatment in decreasing pain and disability [36,37,38]. Therefore, future clinical trials should include clinical populations for assessing the efficacy of visual RUSI biofeedback for facilitating MCE comprehension, performance and retainment compared with other biofeedback methods.

Finally, there are some limitations of the current systematic review. First, we have only included articles written in English; so, we may have missed some relevant studies published in other languages. Furthermore, we did not include those studies which were unpublished. Secondly, due to the variability of the MCE procedures and in the outcomes, a meta-analysis could not be conducted.

## 5. Conclusions

This systematic review found that visual RUSI biofeedback is more effective than common tactile and/or verbal biofeedback for improving MCE performance and retention success in healthy people. There were no clinically important differences between RUSI and pressure unit biofeedback. More high-quality studies with consistent procedures and clinical populations are needed to confirm these findings.

## Figures and Tables

**Table 1:** Methodological quality assessment of the included studies.

Reference.	Study Type	PEDro Scale Items											Score
		1	2	3	4	5	6	7	8	9	10	11	
De la Fuente et al., 2020 [22]	RCT	+	+	−	+	−	−	+	+	+	+	+	7
Henry et al., 2005 [23]	RCT	+	+	−	+	−	−	+	+	+	+	−	6
Herbert et al., 2008 [24]	RCT	+	+	+	+	−	−	+	+	+	+	−	7
Lee et al., 2016 [25]	RCT	+	+	−	+	−	−	−	+	+	+	−	5
Lee et al., 2018 [26]	RCT	+	+	−	+	−	−	+	+	+	+	−	6
Lin et al., 2021 [27]	RCT	+	+	−	+	−	−	−	+	+	+	−	5
McKenna et al., 2020 [28]	RCT	+	+	+	+	−	−	+	+	+	+	+	8
Park et al., 2011 [29]	CT	+	−	−	+	−	−	−	+	+	+	−	4
Solomon et al., 2003 [30]	RCT	+	+	+	+	−	−	+	+	+	+	−	7
Teyhen et al., 2006 [31]	RCT	+	+	+	+	−	+	+	+	+	+	+	9
Van et al., 2006 [32]	RCT	+	+	+	+	−	−	+	+	+	+	−	7

RCT: Randomized Clinical Trial; CT: Clinical Trial. 1: selection criteria; 2: random allocation; 3: concealed allocation; 4: similarity at baseline; 5: subject blinding; 6: therapist blinding; 7: assessor blinding; 8: >85% measures for initial participants; 9: intention to treat; 10: between-group statistical comparisons; 11: point and variability measures. None of the selected articles had a conflict of interest; −: No; +: Yes.

**Table 2:** Data of the studies investigating RUSI as the biofeedback method for MCE.

Study	Population	Comparator	Interventions	Tasks	Muscles Assessed	Outcomes	Results
De la Fuente et al., 2020 [22]	*n* = 20 healthy participants (7M/13F) Age: 25 ± 5 years. Height: 166 ± 10 cm. Weight: 64 ± 6 kg. BMI: 22.2 ± 5.8 kg/m^2^	Visual biofeedback (RUSI) vs. Verbal biofeedback	Participants were placed in a supine position (45° of hip flexion, 90° of knee flexion, the arms close to the trunk in a comfortable position, and the forearms in pronation). Both groups were instructed about the protocols during 5 min before the experiment, using a video. RUSI group watched echography images and were advised to pay attention to the changes in thickness of the TrA. Verbal biofeedback group paid attention to the perception of contraction in the muscles	Four repetitions of the AHE (sustaining an abdominal contraction lasting 7 s after 1 cycle of full inspiration and expiration), with 2 min of rest between repetitions. One basal measure + 3 measures with biofeedback.	Transversus Abdominis	*Normalized Thickness:* Difference between the measurement from each repetition and the basal measure, divided by the basal condition, and expressed in arbitrary units. *Normalized Pressure:* Difference of pressure between each repetition and the basal measure, divided by the basal condition, and in arbitrary units.	Post hoc power = 0.804. Group differences were found (*p* = 0.006) without interactions (*p* = 0.994) or repetition effects (*p* = 0.468). RUSI feedback resulted in larger changes in thickness than the verbal feedback alone (*p* < 0.05). The bias between thickness and pressure for feedback with and without ultrasonography was 0.0490 and −0.0080 respectively. Significant correlation was not found between pressure measurement and thickness. The lowest minimal detectable changes were achieved by using the ultrasonography feedback.
Henry et al., 2005 [23]	*n* = 48 healthy participants (6M/42F) Age: 21.3–23.1 years. Height: 1.7 ± 0.1 m. Weight: 62.5–64.0 kg. BMI: 22.2 ± 5.8 kg/m^2^	Visual Feedback (RUSI) vs. Minimal verbal Feedback vs. Common clinical feedback (verbal descriptive feedback of any observed substitution patterns, verbal corrective feedback, and cutaneous feedback from palpation)	Participants were placed in a supine position with hips flexed between 40° and 80° and knees flexed between 60° and 120°. All groups received instruction in how to perform an AHE. Feedback was given after the first trial and after every other trial thereafter. If the subject appeared to be having difficulty performing the AHE, then the verbal corrective feedback also included a rewording of the instructions to promote understanding.	Each subject was given 2 warm-up trials of the AHE, followed by 10 trials of the AHE, which were assessed as correct or incorrect. Subjects able to perform 3 consecutives correct AHEs on the retention test, as in the initial test, were considered to have retained the ability to perform the AHE correctly.	Transversus Abdominis Internal Oblique External Oblique	Number of trials needed for an individual to consistently perform an AHE. Subjects’ ability to retain the correct performance of the AHE up to 4 days later.	The ability to perform the AHE differed among groups (*p* < 0.001). During the initial session, 12.5% of subjects in verbal feedback group, 50.0% of subjects in common clinical feedback group, and 87.5% of subjects in RUSI group were able to perform 3 consecutive AHEs. There was a difference among groups in the mean number of trials until performance criterion was reached (*p* = 0.0006). No differences were noted among feedback groups with regard to the proportions of subjects able to reach the retention criterion.
Herbert et al., 2008 [24]	*n* = 28 healthy participants (9M/19F) Age: 28 ± 8 years. BMI: 24.0 ± 0.7 kg/m^2^	Constant feedback vs. Variable feedback	Participants were positioned prone on the treatment table with the hips in the neutral position Real-time RUSI of the multifidus muscle at the level of S1 was recorded, transferred to the video recording system, and projected on the television monitor to provide visual feedback. Constant feedback group received visual feedback of the real-time RUSI of successful or unsuccessful multifidus muscle activation on the monitor, but were not given verbal feedback. Variable feedback group received delayed feedback after performing a number of repetitions of the exercise, based on a pre- determined schedule.	Subjects attended 15-min exercise training sessions in the laboratory, twice a week, for a total of 8 training sessions. Participants were asked to recruit the multifidus muscle without extraneous movements and to hold each contraction for 3 s. It also informed the subjects that the training session would consist of 12 repetitions of the exercise and that a successful performance outcome was visualization of muscle movement on the monitor.	Lumbar multifidus muscle	*Performance success:* Defined as isolated isometric recruitment of the first sacral level (S1) multifidus muscle without substitution of extraneous movements such as Valsalva, pelvic tilt, arching the back, lifting the upper trunk, or lifting the lower extremity. *Retention success:* Each subject returned after 1 and 4 weeks. Same procedures were repeated, except that no augmented feedback was provided.	Both groups had similar performances of multifidus muscle recruitment (*p* = 0.26). Constant feedback group had good success (80%) that was maintained at session 8 (84%), with no difference between sessions 1 and 8 (*p* = 0.19). Variable feedback group gradually increased success between sessions 1 and 8 (*p* = 0.002). Both groups sustained their session 8 success when tested for short-term retention at 1 week (Both, *p* > 0.36). At the long-term retention test, the variable feedback group outperformed the constant feedback group (*p* = 0.04), indicating superior motor learning.
Lee et al., 2016 [25]	*n* = 30 healthy participants Age: 20.3–21.1 years Height: 1.66–1.67 m Weight: 55.3–57.0 kg	Visual biofeedback (RUSI) vs. Pressure biofeedback unit vs. Basic training	Participants were placed in a crooked lying position with their knees flexed to 90°. Basic training group received verbal and manual contact biofeedback. Pressure biofeedback group were told to maintain the manometer at 10 mm Hg, starting from 40 mm Hg. RUSI group received training with monitoring of possible contraction of their muscles in the screen.	All of the subjects received AHE training for 15 min. After training, the subjects were measured three times being at rest in a supine position and performing the AHE with which they were trained.	Transversus Abdominis Internal Oblique External Oblique	Thickness measured with ultrasound imaging.	All the groups showed greater TrA thickness (*p* < 0.01) but no changes in IO nor EO (*p* > 0.05). During AHE, the thickness of the musculus transversus abdominis differed significantly among the groups (*p* < 0.05). No significant differences were observed between the basic training and the pressure biofeedback groups, and between the pressure biofeedback and the RUSI groups (*p* > 0.05). However, significant differences between basic training and RUSI were found for TrA (*p* < 0.05). No significant difference was observed among the three groups regarding the thicknesses of the internal oblique abdominal and external oblique abdominal muscles during AHE (*p* > 0.05).
Lee et al., 2018 [26]	*n* = 20 healthy participants Age: 29.0 ± 3.0 years BMI: 22.1 ± 1.7 kg/m^2^	Conventional feedback vs. Visual feedback (RUSI)	Subjects were placed in a supine hook-lying position. Subjects in conventional feedback group were trained AHE using verbal and tactile feedback. Subjects in RUSI group, in addition to the initial education about the conventional feedback, were educated about visual feedback provided with real-time ultrasound imaging.	All subjects received education session about AHE with conventional (verbal and tactile) feedback for 30 min. After the session, the baseline assessment of the muscle activity during AHE was recorded using the surface electromyogra- phy.	Transversus Abdominis Internal Oblique External Oblique	*Ultrasonography* Thickness measurement of the 3 muscles. *Electromiography* Percentages of maximal voluntary contraction were calculated by normalization with maximal voluntary contraction to evaluate how efficiently TrA-IO muscles were activated. Maximal voluntary contraction values of TrA-IO were obtained by maximally twisting upper-body to ipsilateral side against physiatrist’s manual resistance.	After 2 weeks of AHE training, the thicknesses of TrA, IO, and EO muscles in resting were not significantly changed in both groups. Thicknesses of contracted TrA and IO muscles during AHE were significantly increased than those of resting state in both of real-time ultrasound imaging and conventional feedback group (*p* < 0.05). The difference between resting and contraction of TrA muscle thickness in real-time ultrasound imaging feedback group was significantly higher than conventional feedback group (*p* < 0.05), but no for IO (*p* > 0.05). Root mean squares and maximal voluntary contraction values in TrA-IO increased without statistical significance in both groups (*p* > 0.05). The difference in maximal voluntary contraction value of TrA-IO was significantly higher in RUSI group than conventional feedback group (*p* < 0.05). The ratio of root mean squares values of TrA-IO/EO muscles was significantly higher in RUSI group.
Lin et al., 2021 [27]	*n* = 40 healthy participants (9M/31F) Age: 25.9–26.6 years Height: 1.62–1.63 m Weight: 55.6–56.2 kg BMI: 21.0–21.0 kg/m^2^	Verbal biofeedback vs. Visual feedback (RUSI)	During contraction, subjects in the experimental group were required to watch the real-time ultrasound imaging and maintain continuous contraction with maximum effort. Images of the right LM at rest and during maximum isometric contraction were acquired. Images of the right TrA muscle were acquired at rest and during the ADIM maneuver.	All participants were firstly given a verbal explanation regarding the purpose and operation procedure of the experiment and the anatomical structure and function of the muscles before the test. Image acquisition for each condition and each time point (Trest, Tc-max, Tc-15 s, Tc-30 s) was repeated three times.	Lumbar Multifidus Transversus Abdominis	*Lumbar multifidus thickness* Three separate resting ultrasound images were collected immediately after ex- halation *TrA Thickness* ADIM was used to assess the altered muscle thickness associated with a voluntary contraction of the TrA muscle.	No significant differences were found in the thickness of LM at rest (*p* > 0.999), Tc-max (*p* > 0.999), and T15 s (*p* = 0.414) between the two groups. The ability to recruit LM muscle contraction differed between groups at T30 s (*p* = 0.006), with subjects in the experimental group that received visual ultrasound biofeedback maintaining a relative maximum contraction. No significant differences were found in the TrA muscle thickness at rest (*p* > 0.999) and Tc-max (*p* > 0.999) between the two groups. Significant differences of contraction thickness were found at T15 s (*p* = 0.031) and T30 s (*p* = 0.010) between the two groups during the ADIM, with greater TrA muscle contraction thickness in the experimental group.
McKenna et al., 2020 [28]	*n* = 27 patients with unilateral subacromial pain (15M/12F) Age: 54.4–56.8 years BMI: 24.6–29.5 kg/m^2^ NPRS score: 1.0–2.0	Manual facilitation vs. Manual facilitation + RUSI	Participants performed all interventions in the supine position. Participants received individual training in either activating the SA using RUSI feedback with manual facilitation or training with manual facilitation only at the first session. At the second session, the participant received the intervention they did not receive on the first session.	Five practice serratus punches were performed continuously at an approximate speed of 3 s per punch with the participant cued to “reach up”. One minute of rest was then allowed, followed by a further 10 intervention repetitions with ongoing verbal cueing and encouragement, for a total of 15 repetitions during intervention.	Serratus anterior	*Electromiography* Levels of SA activation (normalized to a maximal voluntary isometric contraction).	The predicted marginal mean difference between interventions was 55.5% (95% CI = 13.9% to 97.1%) (*p* = 0.009), favoring the addition of RUSI feedback.
Park et al., 2011 [29]	*n* = 42 healthy males Age: 22.6–23.2 years Height: 1.75–1.76 m Weight: 67.8–67.9 kg BMI: 21.8–22.2 kg/m^2^	RUSI feedback vs. No feedback	Participants were placed in 4 different positions. The experimental group performed AHE with RUSI feedback. The control group performed AHE with no RUSI feedback.	All the subjects were familiarized with AHE with a 30-min training. Measurements were conducted 3 times in each position with 2-min resting between measurements.	Transversus Abdominis Internal Oblique External Oblique	*Ultrasound imaging* Thickness differences between rest and AHE were compared between the two groups.	The difference in internal IO thickness changes between the groups were significant. The differences in EO thickness changes were only significant among the positions. A post hoc analysis of the differences in EO thickness changes among the positions found significant differences between the crook lying and four-point kneeling positions. The TrA thickness changes showed significant interaction between group and position.
Solomon et al., 2003 [30]	*n* = 120 patients with mild to moderate fecal incontinence with at least mild neuropathy (13M/107F) Age: 62.0 ± 12.8 years Exercise compliance: 83.0%	Digital examination feedback vs. Transanal RUSI vs. Anal manometry	All patients were lying in the left lateral position. In the digital examination group, patients performed a full set of supervised exercises guided by digital per anal examination of the external sphincter. In the RUSI group, patients were taught how to contract the anal sphincters while watching the real-time ultrasound display on the monitor screen, and a full set of exercises were performed during each treatment session. In the anal manometry group, Patients were taught how to contract and relax the anal sphincters while attending to the pressures generated in the anal canal, and a full set of exercises were performed during each treatment session.	All participants performed a full set of exercises, consisting of ten five-second sphincter contractions, each at one-second intervals, repeated ten times (a total of 100 contractions). All patients were urged to perform an identical set of exercises twice per day between outpatient visits and were asked to estimate the percentage of exercises they had actually completed.	Pelvic floor	St. Mark’s Hospital fecal incontinence score Pescatori fecal incontinence score Patient’s self-assessment of fecal incontinence severity using a visual analog scale Investigator’s assessment of fecal incontinence severity using a visual analog scale. Quality-of-life measure using Direct Questioning of Objectives Resting and maximal squeeze anal canal manometric pressures Isotonic fatigue time Isometric fatigue contractions	One hundred two patients (85 percent) completed the four-month treatment program. Across all treatment allocations, patients experienced modest but highly significant improvements in all nine outcome measures during treatment, with 70 percent of all patients perceiving improvement in symptom severity and 69 percent of patients reporting improved quality of life. With the possible exception of isotonic fatigue time, there were no significant differences between the three treatment groups in compliance, physiologic sphincter strength, and clinical or quality-of-life measures. Correlations between physiologic measures and clinical outcomes were much stronger with ultrasound-based measures than with manometry.
Teyhen et al., 2005 [31]	*n* = 30 patients with chronic low back pain (18M/12F) Age: 62.0 ± 12.8 years Exercise compliance: 83.0%	Tactile and verbal feedback vs. Tactile, verbal and RUSI feedback	All patients were placed on quadruped position. In both groups, tactile and verbal instructions were provided to all subjects in each position. After the training in quadruped, patients were then randomly assigned to receive further instruction using traditional training (visual + tactile feedback) or traditional training with biofeedback in the ADIM.	To determine the baseline performance of the patient’s ability to per- form the ADIM prior to training, subjects were instructed to contract their abdominals by bringing their belly button up and in towards their spine. No other instruction or tactile cues were provided. After baseline measurements were obtained, all subjects received an education session and training in the ADIM in 3 positions: quadruped, seated and supine. A total of 5 contraction attempts, each with a 10-s hold, were performed in each of the 3 positions.	Transversus Abdominis Internal Oblique External Oblique	*Ultrasound imaging* Thickness differences between rest and ADIM. In addition, a reliability analysis was performed. *Performance retention* At the end of the first session, all subjects received instruction on the home exercise program and were asked to return after 4 days.	Intrarater reliability measuring lateral abdominal muscle thickness exceeded 0.93. On average, patients in both groups demonstrated a 2-fold increase in the thickness of the TrA during the ADIM. Performance of the ADIM did not differ between the groups.
Van et al., 2006 [32]	*n* = 25 healthy participants (6M/19F) Age: 19.1–19.9 years	Verbal feedback vs. Verbal and RUSI feedback	Subjects were placed in a prone position. All subjects received feedback on the number of millimeters of increase in muscle thickness that occurred with contraction of the multifidus (KR), with the aim being to increase this value. In addition to the provision of KR, subjects in the other group received biofeedback in the form of visual observation of the ultrasound image of the muscle contraction as it occurred.	Prior to testing in the acquisition phase, all subjects received the same initial explanation relating to the multifidus muscle. Each subject performed a total of 10 contractions (acquisition phase) with 20 s of rest between measurements. After completing the 10 trials in the acquisition phase, all subjects were asked to return in 1 week for follow-up assessments (retention phase).	Lumbar multifidus	*Ultrasound imaging* To assess multifidus muscle contraction, the difference between the multifidus muscle thickness at rest and during contraction was calculated.	Subjects from both groups improved their voluntary contraction of the multifidus muscle in the acquisition phase (*p* < 0.001) and the ability to recruit the multifidus muscle differed between groups (*p* < *0*.05), with subjects in the group that received visual ultrasound biofeedback achieving greater improvements. In addition, the group that received visual ultrasound biofeedback retained their improvement in performance from week 1 to week 2 (*p* > 0.90), whereas the performance of the other group decreased (*p* < 0.05).

ADIM: Abdominal Draw-In Maneuver; AHE: Abdominal Hollowing Exercise; EO: External Oblique; IO: Internal Oblique; LM: Lumbar Multifidus; TrA: Transversus Abdominis.

## Data Availability

The data that support the findings of this study are available from the corresponding author (J.A.V.-C.), upon reasonable request.
